# Supplementary material for: Quantifying brain state transition cost via Schrödinger Bridge
Source: Netw Neurosci. 2022 Feb 1;6(1):118–34. doi: 10.1162/netn_a_00213 (PMC8959122; doi:10.1162/netn_a_00213)
Supplement: Supplementary file 1 [file netn-06-118-s001.pdf]

Kawakita, G., Kamiya, S., Sasai, S., Kitazono, J. & Oizumi., M.(2022). Supporting information for "Quantifying brain state transition cost via Schrödinger Bridge." *Network Neuroscience*, *6*(1), 118–134. https://doi.org/10.1162/netn_a_00213

S1: Robustness with different numbers of clusters

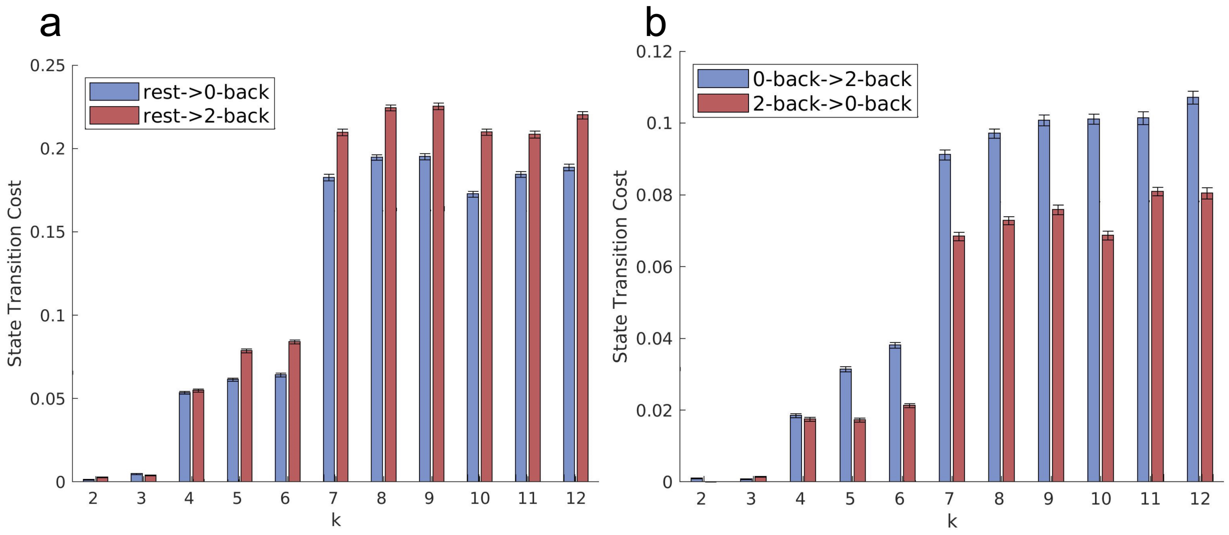

Figure 1: (a) Brain state transition cost from the resting state to 0-back and 2-back tasks. For $k \geq 4$, the cost for transitioning to the 2-back task is larger than that of the 0-back task, which is consistent with the results in the manuscript with $k = 8$ (Figure 3a). (b) Brain state transition cost between the 0-back task and 2-back task. For $k \geq 4$, the cost for transitioning from the 0-back task to the 2-back task is larger than that of the opposite direction. This is in line with the result in the main text with $k = 8$ (Figure 4a). The results in (a) and (b) indicate the robustness of the methodology used in the manuscript with respect to the number of clusters for k-means clustering.

S2: Order of the magnitudes of transition cost from the resting state

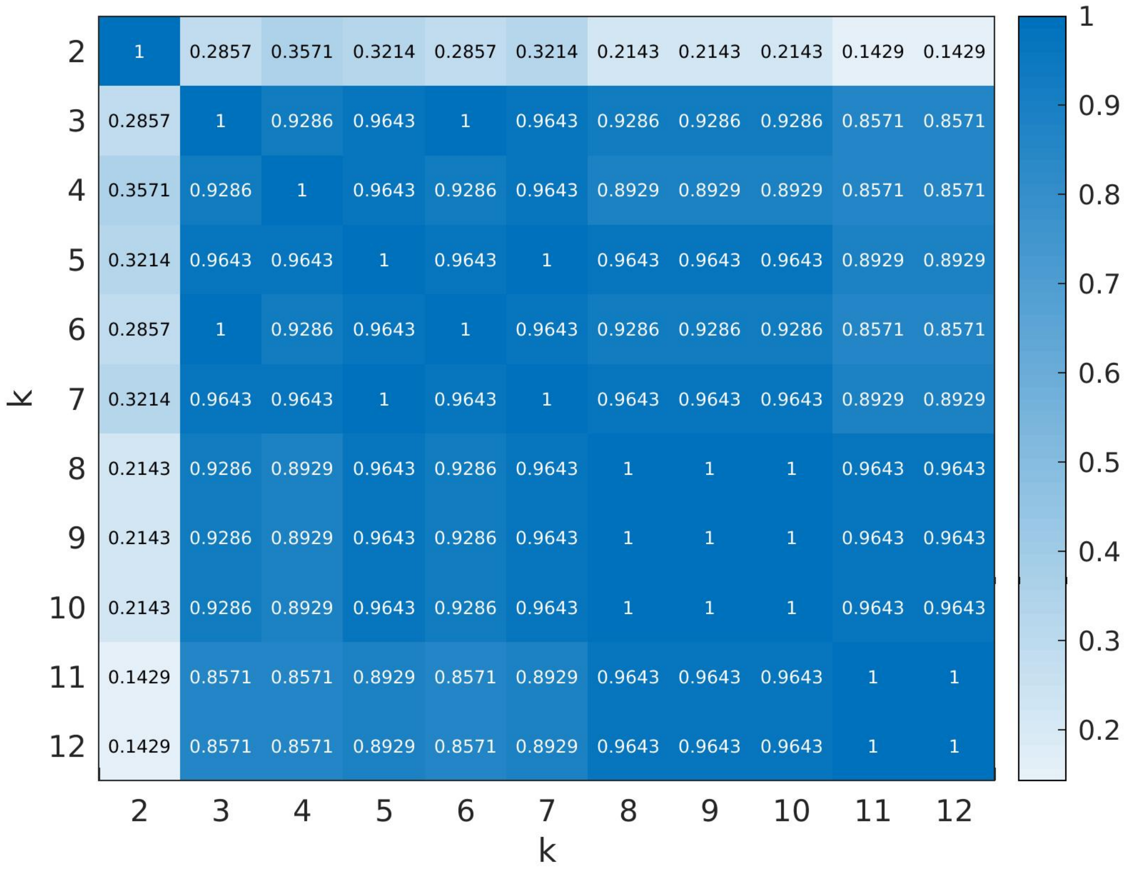

Figure 2: Spearman's rank correlation coefficients between the order of magnitudes of transition cost from the resting state for different numbers of clusters. For $2 \leq k \leq 12$, we computed the Spearman's rank correlation coefficient between the order of magnitude of transition cost from the resting state to the seven cognitive tasks (emotion, gambling, language, motor, relational, social, and working memory) for different numbers of clusters. The figure shows that the order is highly consistent for $k \geq 3$, which demonstrates the robustness of the results in the manuscript (Figure 3b).

S3: Asymmetry of brain state transition cost

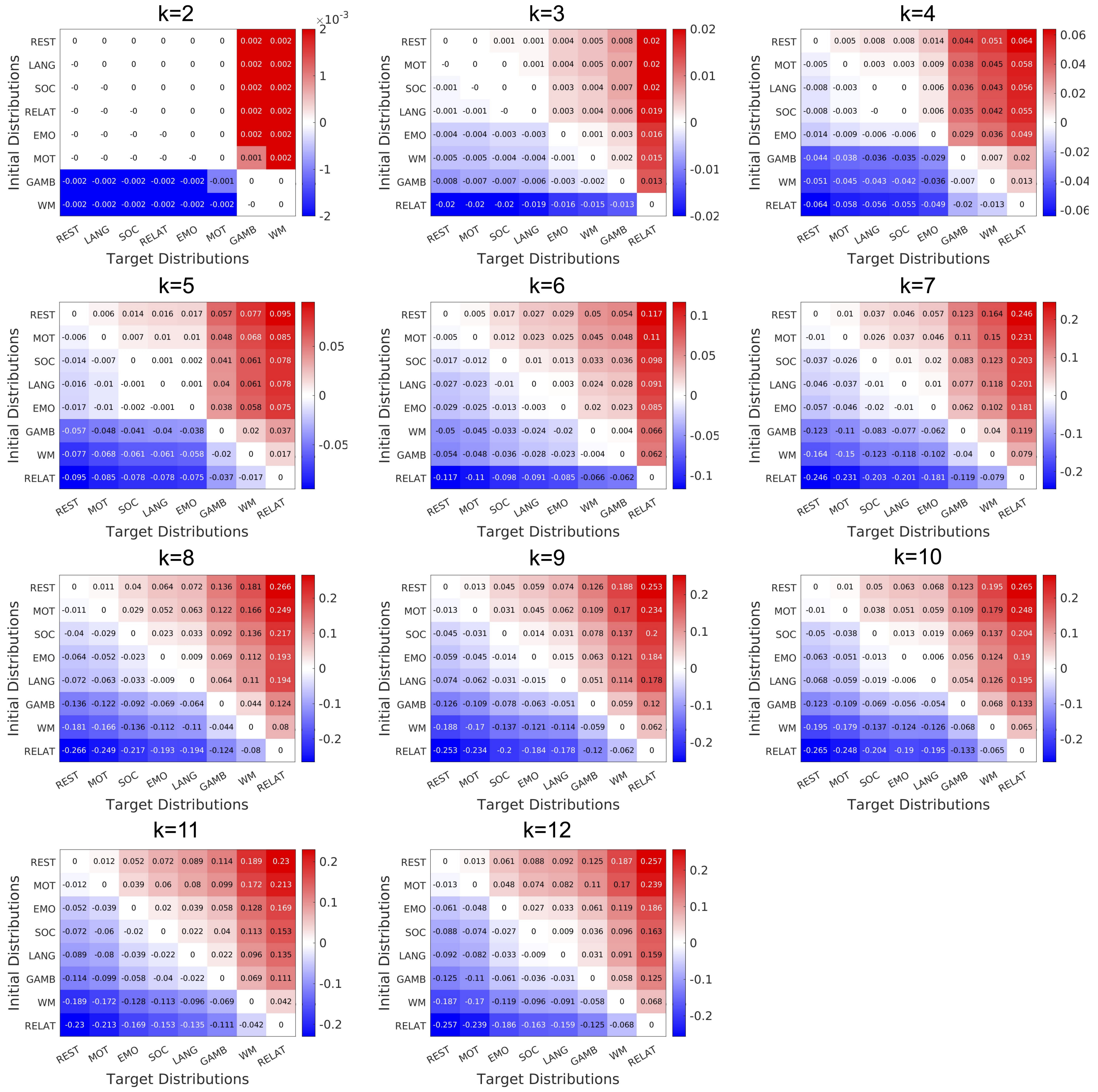

Figure 3: Asymmetry of brain state transition cost for different numbers of clusters. We tested whether the asymmetric relationship for transition costs between eight tasks, including the resting state (emotion, gambling, language, motor, relational, social, and working memory) (see Results for the definition) may hold for $k \neq 8$. As shown in the figure, we observed the asymmetry of brain state transition cost between all pairs of tasks for $2 \leq k \leq 12$.

S4: Criteria for determining the number of clusters for k-means clustering algorithm

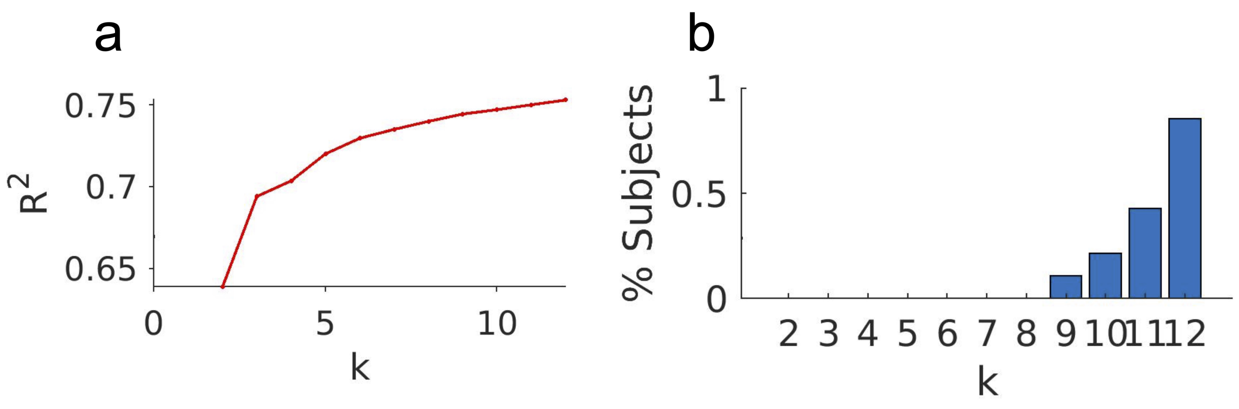

Figure 4: (a) The percentage of variance explained by the number of clusters varying from $k = 2$ to $k = 12$. We observed that the explained variance plateaued around 75% after $k = 5$. (b) The percentage of subjects missing a cluster (brain state) from one task session. For $k > 8$, we observed that some clusters were missing from some subjects. Based on the results in (a) and (b), we set the number of clusters to $k = 8$ for the analysis in the manuscript. However, as shown in S1-S3, our main results are robust for $4 \leq k \leq 12$.

S5: Figures of brain maps

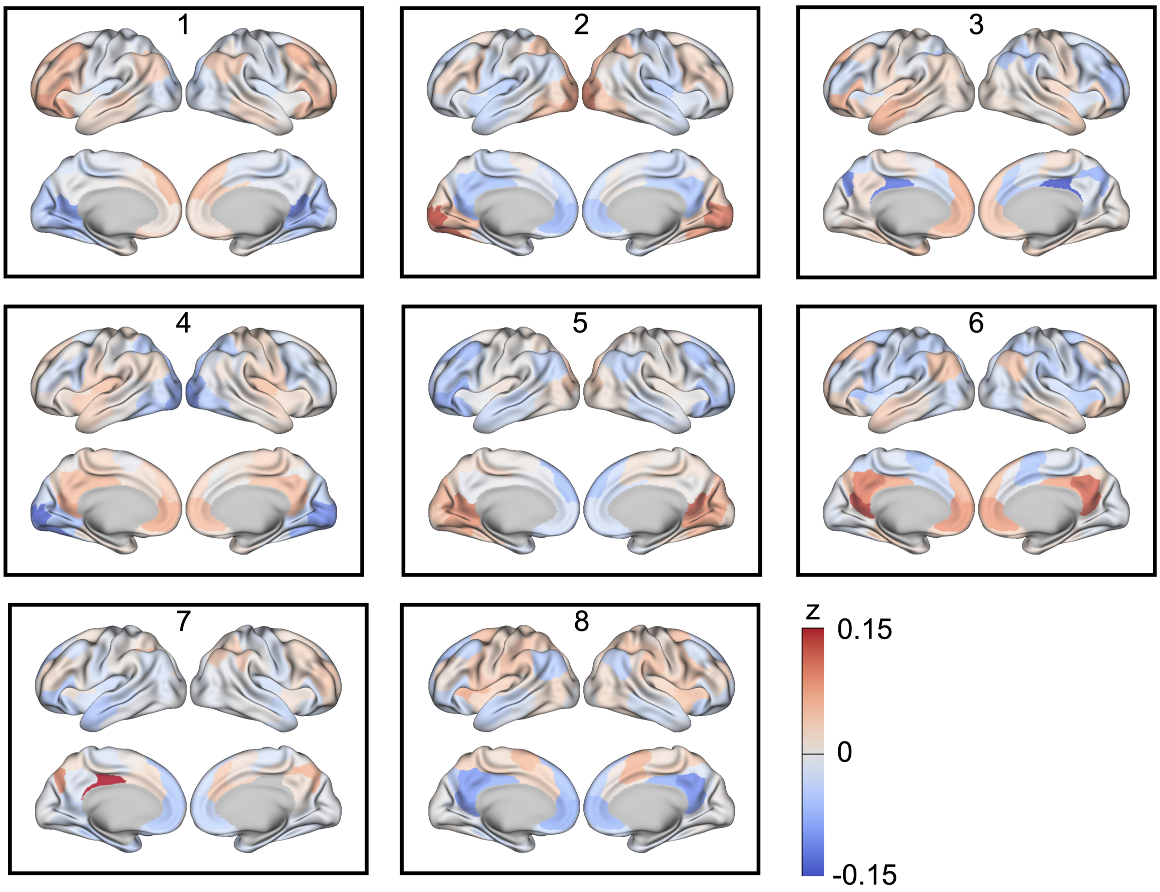

Figure 5: The z-scored activities of eight brain states defined by the centroids of eight clusters determined by k-means clustering algorithm. Brain regions with high-amplitude activities are colored red and those with low-amplitude activities are colored blue.

S6: Reproducibility of the results with $T > 1$

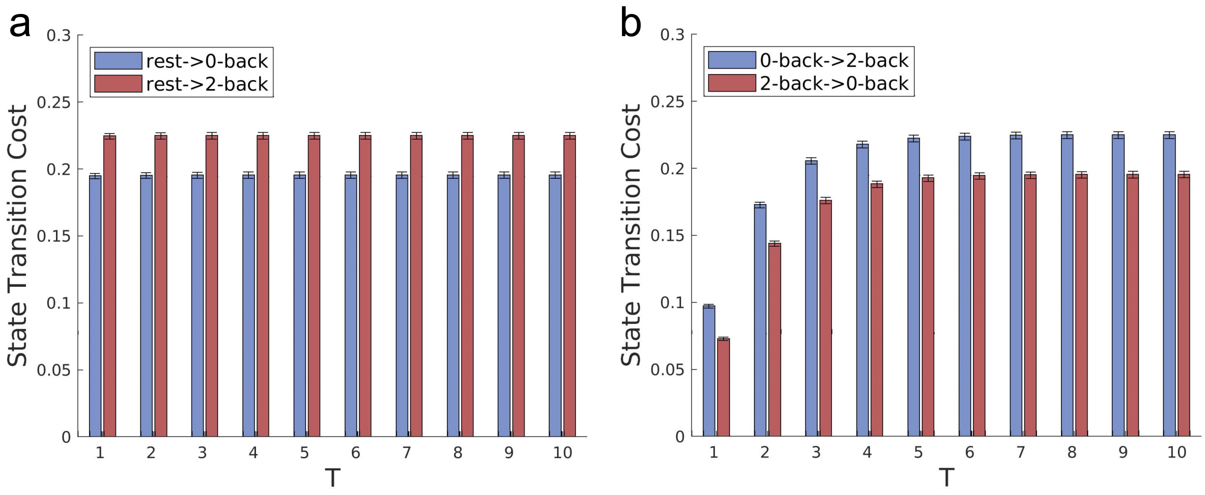

Figure 6: Brain state transition cost when the time horizon, T, is set to $T > 1$. **(a)** Brain state transition cost from the resting state to the 0-back and 2-back tasks with T ranging from 1 to 10. Increasing T does not affect the degree of the transition cost because the initial distribution is set to be the probability distribution of the resting state, which is the stationary distribution of the transition probability of the uncontrolled path. **(b)** Brain state transition cost between the 0-back and 2-back tasks. Increasing T makes the cost larger up to $T = 4$, but the degree of cost stays the same for $T > 5$, as the endpoint distribution of the uncontrolled path, $q(X_T)$ converges to the stationary distribution of the resting state transition probability as T becomes larger.
